# Commentary: Self-management, self-efficacy and knowledge among patients under haemodialysis: a case in Iran

**DOI:** 10.1177/1744987120904902

**Published:** 2020-03-14

**Authors:** Alison F Wood

**Affiliations:** Lecturer, Edinburgh Napier University, Scotland, UK

Papers that explore elements of haemodialysis treatment continue to be relevant to current practice and healthcare practitioners. Haemodialysis as a lifesaving treatment option for patients with end-stage kidney failure continues to be accessible across the world for patients who await, or are not eligible for, kidney transplant. As chronic kidney disease and end-stage kidney failure remains prevalent across the world, exploring elements of this treatment and how patients can be better supported through this process is a vital element for continuing research.

This paper presents key elements to consider when supporting patients who receive haemodialysis in Iran. A greater understanding of predictors of self-management allow healthcare professionals to engage in supportive practices that could improve self-management in this patient population. The study covered four haemodialysis centres and 159 patient participants in Iran, allowing a large sample size. Collecting demographic data alongside the three well-established questionnaires adds strength to the dataset. The use of validated scales within this population contributes to the use of these questionnaires, both within Iran and other countries.

A greater understanding of the complexities of the Iranian health system and culture and the potential impact to the results would have added strength to the transferability of the results to other cultures with similarities. Some of the demographic data related to the self-management and self-efficacy should be explored to support practitioners in caring and supporting these patients with varying requirements.

Haemodialysis units are complex, they provide life-saving treatment regularly and become a key part of a patient’s life. Considering ways in which a patient’s quality of life can be improved both within and outside the haemodialysis unit requires constant consideration. Too often do healthcare professionals consider only the treatment presented to them rather than the proactive elements to change and support patients with their life and managing the condition they have.

Improvements in self-management can both enhance quality of life and reduce mortality and side effects; any of these are positives for any haemodialysis patient. Although self-management is only one element for this patient population, its relationship to quality of life is known and understanding this better can only lead to better care for our patients.

**Alison F Wood** is a Lecturer in the School of Health and Social Care, Edinburgh Napier University. She is the Programme Leader for non-medical prescribing and a registered adult nurse. Her PhD and most recent clinical work experience have focused on haemodialysis nursing care within a haemodialysis outpatient department. Other scholarly interests include the use of ethnographic methodology and patient care experiences as well as nurse education related to biosciences and the development of independent prescribing. She is part of the centre for Cardiovascular Health at Edinburgh Napier University and holds an Honorary Nurse Consultant post within NHS Lothian, Scotland.

